# Movement Recognition and Muscle Force Estimation of Wrist Based on Electromyographic Signals of Forearm

**DOI:** 10.3390/bios15040259

**Published:** 2025-04-17

**Authors:** Leiyu Zhang, Zhenxing Jiao, Yongzhen Li, Yawei Chang

**Affiliations:** 1 Beijing Key Laboratory of Advanced Manufacturing Technology, Beijing University of Technology, Beijing 100124, China; zhangleiyu@bjut.edu.cn (L.Z.); jiaozhenxing0109@163.com (Z.J.); 2 Institute for Smart Ageing, Beijing Academy of Science and Technology, Beijing 100089, China

**Keywords:** wrist rehabilitation robot, machine learning, movement recognition, muscle force estimation

## Abstract

To enhance wrist impairment rehabilitation efficiency, self-rehabilitation training using healthy-side forearm sEMG was introduced, improving patient engagement and proprioception. A sEMG-based movement recognition and muscle force estimation algorithm was proposed to transmit the estimated results to a wrist rehabilitation robot. Dominant eigenvalues of raw forearm EMG signals were selected to construct a movement recognition model that included a BPNN, a voting decision, and an intensified algorithm. An experimental platform for muscle force estimation was established to measure sEMG under various loads. The linear fitting was performed between mean absolute values (*MAV*s) and external loads to derive static muscle force estimation models. A dynamic muscle force estimation model was established through linear fitting average *MAV*s. Volunteers wore EMG sensors and performed six typical movements to complete the verification experiment. The average accuracy of only BPNN was 90.7%, and after the addition of the voting decision and intensified algorithm, it was improved to 98.7%. In the resistance training, the measured and estimated muscle forces exhibited similar trends, with RMSE of 4.2 N for flexion/extension and 5.8 N for ulnar/radial deviation. Under two different speeds and loads, the theoretical and estimated values of dynamic muscle forces showed similar trends with almost no phase difference, and the estimation accuracy was better during flexion movements compared to radial deviations. The proposed algorithms had strong versatility and practicality, aiming to realize the self-rehabilitation trainings of patients.

## 1. Introduction

The wrist joint, as a complex articular structure composed of multiple joints, plays a crucial role in human daily life. However, it is also one of the most susceptible joints to injury. The main causes of wrist impairments include stroke, accidental falls, and traffic accidents [1], which not only severely affect the quality of life of patients but also impose a heavy economic burden on their families [2,3]. An early initiation of rehabilitation therapy could significantly enhance the effectiveness of recovery and expedite the restoration of normal function [4,5]. Traditional manual rehabilitation is often time-consuming and labor-intensive [6,7,8], and the assessment of recovery is primarily based on physicians’ subjective consciousness, lacking corresponding physiological muscle indicators [9]. As a result, it has been difficult to meet the personalized and differentiated rehabilitation needs of patients. In recent decades, with the continuous advancement of robotics and artificial intelligence technologies, rehabilitation robots have demonstrated significant positive effects in the fields of stroke, spinal cord injury, and motor dysfunction. Furthermore, the integration and innovation of rehabilitation robots with various technologies have further enhanced their efficiency and accuracy [10]. Cappello et al. [11] developed a wrist rehabilitation robot integrated with a virtual reality environment, capable of providing visual feedback during rehabilitation training. The robot can assess patients’ proprioception, collect physiological data during the rehabilitation process, and monitor the progress of rehabilitation. Spencer et al. [12] integrated electronic games into a wrist rehabilitation robot (CRAMER) to enhance patient engagement and enjoyment. Andrikopoulos et al. [13] designed another robot (EXOWRIST) based on pneumatic muscles, capable of facilitating wrist joint flexion/extension (F/E) and radial/ulnar deviation (R/U). This device possesses the advantages of lightweight nature, high safety, and good compliance. Parallel, serial, or flexible wrist rehabilitation robots have become a research focus and possess many mature devices [14]. Combining surface electromyography (sEMG) and artificial intelligence is an important direction for future development. sEMG signals are a kind of bioelectrical signal generated during muscle activities and are recorded by electrodes placed on the skin surface. As a bioelectrical control signal, they can be used to control devices such as prosthetic limbs and exoskeletons, enabling natural human–machine interaction.

With the continuous advancement of artificial intelligence technology, numerous scholars have progressively shifted their research focus toward exoskeleton control based on sEMG signals [15,16]. sEMG-based control algorithms can better emphasize patient participation in the rehabilitation process, which significantly enhances proprioception and training efficiency. Tsujiuchi et al. [17] proposed a real-time forearm EMG-based action recognition method, which could predict seven types of movements with an accuracy rate of over 90%. Xiao et al. [18] proposed an algorithm for estimating angles of elbow joints based on sEMG signals, where this algorithm took the time-domain features of sEMG signals as inputs of a random forest and the elbow joint angles as outputs. The estimated results indicated that the average deviation relative to actual elbow angles was 0.08°, with a standard deviation of 5.01°. Yanchen et al. [19] proposed a deep learning algorithm using sEMG signals to estimate joint angles and complex movements of upper limbs for the three-dimensional motion control of mirror therapy. Fukano et al. [20] utilized convolutional neural networks (CNNs) to construct a model capable of recognizing 53 types of gestures, with an accuracy rate exceeding 60% and a response time within 300 ms. Yang et al. [21] decoded human movements with multiple degrees of freedom (DoF) from sEMG signals using deep learning algorithms, demonstrating good predictive performance across various scenarios. Gelareh et al. [22] employed CNNs for estimating muscle contraction force based on the time-domain and frequency-domain characteristics of high-density electromyography (HD-EMG) signals. Yu et al. [23] proposed a deep learning algorithm based on stacked autoencoders and deep neural networks (SAE-DNN) to estimate wrist torques. This algorithm demonstrated higher accuracy compared to traditional regression methods. Furthermore, an innovative estimation algorithm [24] for wrist torques was established based on the decomposition and image reconstruction of the motor units’ EMG signals. Specifically, this algorithm decoded HD-EMG signals into motor unit spike sequences and reconstructed motor unit-specific images using these spike sequences and their corresponding motor unit action potentials. Yu et al. [25] further proposed a multi-DoF wrist torque estimation method based on instantaneous sEMG images. Although this torque estimation method excelled in accuracy, the constructed model was relatively complex and consumed huge computational resources. Khokhar et al. [26] took time-domain features of EMG signals as inputs for support vector machines, with wrist torques as outputs, demonstrating the feasibility of predicting wrist torque from EMG signals. Furthermore, Khokhar et al. [27] developed a wrist rehabilitation robot controlled by sEMG signals, using a combination of principal component analysis and neural networks to recognize wrist movements. Rechy-Ramirez et al. [28] designed a human-machine interface system for wrist rehabilitation, using leap motion controllers and MYO armbands to recognize five wrist actions (relaxation, extension, pronation, supination, and flexion), thereby controlling the movement of a ball in a game interface. For the muscle force estimation, there are three commonly used models. The first is the Hill model [29] proposed by A.V. Hill, which is a classic physiological model-based algorithm requiring the collection of various physiological parameters to construct different Hill models. However, these physiological parameters are difficult to obtain and the model establishment is also challenging. The second is muscle force estimation models using machine learning [30], and the third involves establishing a mathematical relationship between EMG signals and muscle forces [31]. Hussain [32] et al. provided a comprehensive review of state-of-the-art robotic devices for wrist rehabilitation, highlighting the importance of integrating AI algorithms for better control and personalization of rehabilitation protocols. They discussed various control strategies, including fuzzy logic and neural networks, which have been shown to enhance the adaptability and responsiveness of rehabilitation robots. Saeedi [33] et al. proposed an innovative approach using artificial neural networks (ANN) and adaptive neuro-fuzzy inference systems (ANFIS) for inverse kinematics analysis of a wrist rehabilitation robot. This method demonstrated improved accuracy and efficiency in predicting joint movements, which is crucial for effective rehabilitation. Another notable study by Mashayekhi [34] et al. introduced an EMG-driven fatigue-based self-adapting admittance control strategy for a hand rehabilitation robot. This algorithm adapts to the patient’s muscle fatigue, providing a more personalized and effective rehabilitation experience.

For enhancing the rehabilitation efficiency of wrist impairments, a self-rehabilitation training protocol was proposed to improve patient engagement and proprioception through the utilization of sEMG signals from the healthy forearm. To achieve this, sEMG-based movement recognition and muscle force estimation algorithms were developed, which were intended to transmit the estimated outcomes to a wrist rehabilitation robot, thereby facilitating a more personalized and responsive treatment approach. The methodology involved the collection of raw forearm sEMGs, followed by the selection of dominant eigenvalues to construct a robust movement recognition model. This model was comprised of a backpropagation neural network (BPNN), which served as the foundation for pattern recognition, complemented by a voting decision to enhance classification accuracy. Furthermore, an intensified algorithm was integrated to refine the predictive capabilities, ensuring that the movement recognition was both accurate and reliable. An experimental platform for the muscle force estimation was established to measure sEMG under various loads, and the corresponding mean absolute values (*MAV*s) of the EMG signals were obtained. The linear fitting was performed between *MAV* and external loads to derive static muscle force estimation models for the F/E and R/U movements. A dynamic muscle force estimation model was established through linear fitting average *MAV*s.

## 2. Materials and Methods

### 2.1. Experimental Platform

**Figure 1:**
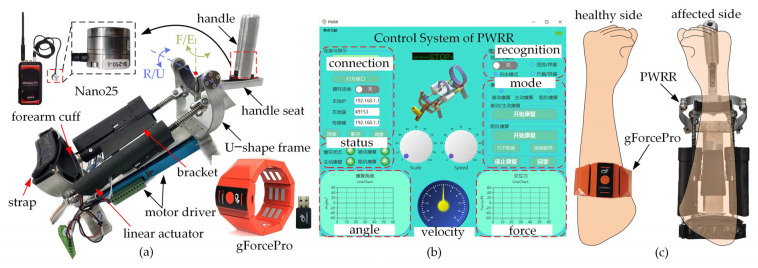
Experimental platform. (**a**) PWRR. (**b**) Interactive interface. (**c**) Rehabilitation scenario.

A parallel wrist rehabilitation robot (PWRR) with a 2-SPU/RR configuration was developed to facilitate motor rehabilitation for wrist F/E and R/U movements where a compact linear actuator was specifically designed [35], as shown in Figure 1a. A six-axis force sensor (Nano25, ATI Industrial Automation, Apex, NC, USA) embedded within the handle and an armband sensor with eight acquisition channels (gForcePro, OYMotion, Shanghai, China) were utilized to collect interactive forces and forearm sEMG signals during the passive and active trainings. The gForcePro with dry electrodes was connected to a PC via Bluetooth, offering the advantages of easy wear and no need for electrode replacements. The affected wrist was placed on the forearm cuff and bracket and bound by a strap. The passive chain RR was installed inside the U-shape frame and a passive prismatic joint in the handle seat to relieve the restraint forces. The motion recognition of the healthy side, the training mode, and kinematic information were displayed on the interactive interface (Figure 1b). A wrist motion recognition algorithm and a muscle force estimation model based on the characteristics of sEMG signals were established to realize self-rehabilitation trainings. The healthy side guided the affected side to move along the predetermined trajectory according to the recognition results of the healthy forearm, significantly enhancing the patient’s proprioception (Figure 1c).

### 2.2. Movement Recognition Algorithm of Wrist

The sEMG signal is the temporal and spatial superposition of motor unit action potentials from numerous muscle fibers, reflecting the intensity of muscle activity. The coordinated movements of limbs are primarily controlled by signals emitted by the α-neurons of the motor central nervous system. These signals are transmitted to the branches of muscle fibers and trigger contraction of the muscle fibers upon appropriate stimulation, thereby driving the muscles to perform predetermined movement tasks [36].

Based on the characteristics of sEMGs, a movement recognition algorithm was established to recognize the type and angle of wrist movements. This algorithm consisted of a BPNN, a voting algorithm, and an enhancement algorithm to approve the classification accuracy. A dataset was necessary for a classification model of wrist movements. A healthy participant (male, height 172 cm, weight 67 kg) wore gForcePro on the middle of the forearm to collect sEMGs of seven movements: flexion with a small range (F-S), flexion with a large range (F-L), extension with a small range (E-S), extension with a large range (E-L), radial deviation (R), ulnar deviation (U), and relaxation. The duration of each movement was 30 s, and eight channels of sEMGs were collected to form the dataset. It was difficult for the raw sEMG signals to indicate the motion intention directly. Hence, five eigenvalues of the time domain were selected: *MAV*, root mean square (*RMS*), variance (*VAR*), zero crossing (*ZC*), and waveform length (*WL*).

(1) $$\mathrm{MAV}=\frac{1}{N}\sum_{i=1}^{N}\left|x_{i}\right|$$

(2) $$\mathrm{RMS}=\sqrt{\frac{1}{N}\sum_{i=1}^{N}x_{i}^{2}}$$

(3) $$\mathrm{VAR}=\frac{1}{N}{\sum_{i=1}^{N}\left(x_{i}-\frac{1}{N}\sum_{i=1}^{N}x_{i}\right)}^{2}$$

(4) $$\begin{array}{l} \mathrm{ZC}=\frac{1}{N}\sum_{i=1}^{N-1}f\left(x_{i}\right)\\ f\left(x_{i}\right)=\left\{\begin{array}{l} 1\\ 0 \end{array}\right.\begin{array}{c} \;x_{i}x_{i+1}<0\;\mathrm{and}\;\left|x_{i}-x_{i+1}\right|>x_{th}\\ \mathrm{else} \end{array} \end{array}$$

(5) $$\mathrm{WL}=\frac{1}{N}\sum_{i=1}^{N-1}\left|x_{i+1}-x_{i}\right|$$

where *x_i_* is the sEMG signal at the time *i*, and *N* denotes the total number of sEMG signals in the selected duration.

**Figure 2:**
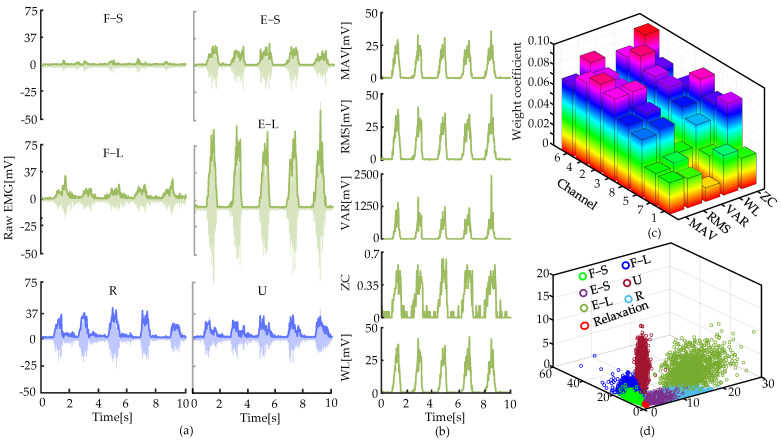
Dataset for a classification model. (**a**) Raw sEMGs of channel 4. (**b**) Five time−domain eigenvalues of the extension motion over a large range. (**c**) Weight coefficients of the eigenvalues. (**d**) Classification of seven movements.

The raw signals from channel 4 during six movements are illustrated in Figure 2a. The eigenvalues of the raw signals of E-L were calculated, as depicted in Figure 2b. The ReliefF algorithm was adopted to select eigenvalues with dominant weight coefficients to eliminate redundant features and enhance the accuracy of the movement recognition algorithm. The ReliefF algorithm is a widely used feature selection method that can effectively handle noisy and incomplete data. There were 40 eigenvalues in total for the eight channels. Then, the weight coefficients of the eigenvalues for each channel were obtained (Figure 2c). Two eigenvalues with maximum weight coefficients of each channel were selected. Hence, 16 eigenvalues were used to construct the dataset. The dataset was dimensionally reduced and projected into a coordinate system via non-negative matrix factorization (NMF), as shown in Figure 2d, demonstrating satisfactory classification performance.

The movement recognition of the wrist was generally categorized as a classification problem. A customizable BPNN was selected. Compared with some traditional algorithms, it had obvious advantages in handling nonlinear and complex problems. In this model, the output *y_i_* was obtained by weighting and summing the inputs of multiple neurons *X_i_*. The error between the output *y_i_* and the actual value was propagated backward to calculate the leading layer error. Through multiple iterations of propagation and weight adjustment, an eventual classification model with minimal error was achieved [37]. The primary structure of this model was comprised of the input layer, hidden layer, and output layer. The forward propagation equation is as follows:

(6) $$y_{i}=g(\sum_{i=1}^{n}w_{i}X_{i}+b)$$

where *g* is the activation function, *w_i_* is the weight of the *i*th input signal, *b* is the bias, and *n* is the number of input eigenvalues.

Two hidden layers were constructed, and the number of neurons *M* in each hidden layer can be determined through an empirical formula:

(7) $$M=\sqrt{m+n}+a$$

where *m* and *n* represent the number of nodes in the input and output layers, respectively, *a*∈[0, 10]. Based on the number of the selected eigenvalues and the recognized actions, the parameters were obtained, where *m* = 16, *n* = 7, and *a* = 10. Then, the number of neurons *M* in the hidden layer could be calculated, where *M* = 15.

As the movement recognition involved multi-class classification, the cross-entropy error function was selected to calculate this error *L* of BPNN:

(8) $$L=-\sum_{i=1}^{N}y_{i}\log({\hat{y}}_{i})$$

where *N* is the number of categories, and ${\hat{y}}_{i}$ denotes the predicted probability vector for each category in this model.

Eighty percent of the dataset established above was utilized as the training set of BPNN, and the remaining 20% was used as the test set. The learning rate was set to 0.001, and the number of iterations was set to 50,000. The recognition accuracy was able to reach 97.4% after the BPNN model was trained sufficiently.

**Figure 3:**
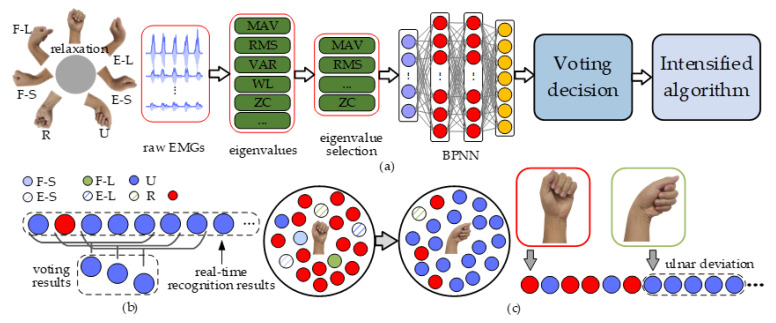
Movement recognition algorithm of healthy wrist. (**a**) Flow chart. (**b**) Voting decision. (**c**) Intensified algorithm.

This trained model was then deployed into the control system of PWRR, as shown in Figure 3a. However, it was difficult to ensure the same wearing position of the armband and the wrist movement during the test state mentioned above and the actual recognition. This difference led to a reduction in the recognition accuracy. To enhance the stability of the movement recognition algorithm, a voting decision was added. For the ulnar deviation, five adjacent recognition results were selected at a time, and the results occurring most frequently were taken as the final output. This process then discarded the last recognition result in the series and added a new recognition result at the front, followed by another round of voting and output (Figure 3b). This voting decision could effectively eliminate erroneous recognitions. To further enhance the recognition accuracy during the rehabilitation process, an intensified algorithm was adopted (Figure 3c). When the wrist initially remained relaxed, the recognition results were a multitude of relaxation. When the wrist completed a movement of ulnar deviation, the recognition results mainly included ulnar deviation. As it was difficult to maintain smooth motion continuity during the aforementioned movements, a slight pause or moving back might lead to wrong recognitions. Fortunately, the incorrect results were a lack of continuity and infrequent occurrence. Hence, the intensified algorithm was simplified: if five consecutive identical results were received, it indicated that the patient wished to perform the corresponding movement.

### 2.3. Muscle Force Estimation Model

Muscle force estimation was used to estimate the force of muscle contraction without a force sensor, which has been widely applied in rehabilitation training and human-computer interaction [38,39]. There existed a positive correlation between the muscle force and sEMG signals [40], and the time-domain information of sEMG signals was also closely related to the muscle force [41]. Hence, a linear regression model was established to estimate the muscle force, with time-domain features used as the main input parameters. This approach exhibited excellent computational efficiency and was therefore well-suited for real-time control.

**Figure 4:**
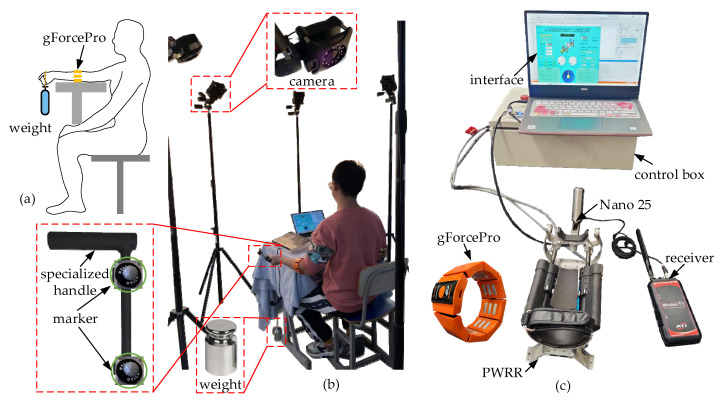
Experimental platform of muscle force estimation. (**a**) Muscle forces under different weights. (**b**) Dynamic experiments under VICON system. (**c**) Performance experimental platform.

An experimental platform for the muscle force estimation was constructed, as shown in Figure 4. Volunteers were required to wear gForcePro with natural posture and grasp a handle suspended with weights. A force sensor (Nano25) was embedded within the handle. A total of four movements (F/E and R/U) were performed, and each movement was conducted for 10 sets. Each set lasted 30 s, with the load starting at 5 N and increasing by 5 N intervals up to 50 N (Figure 4a). After each movement, volunteers rested for 10 min to prevent muscle fatigue. The entire procedure was to be repeated three times. To further explore the influence of wrist movements and external loads on the muscle force estimation, a dynamic experiment was conducted utilizing the VICON optical motion capture system. A specialized handle was designed to facilitate better adherence of markers (Figure 4b). The experiment was divided into four groups: slow flexion (F-slow), fast flexion (F-fast), slow radial deviation (R-slow), and rapid ulnar deviation (U-fast). Each group consisted of three sub-experiments with varying loads (0 N, 10 N, 20 N). In the slow flexion, for example, F-slow-10 denoted the flexion movement of the wrist at a slow speed with a load of 10 N. The volunteers wore gForcePro, and the sEMG signals were collected with the help of the control system of PWRR (Figure 4c). Similarly, the wrist dynamics information (including angle, velocity, acceleration, etc.) was acquired in real time.

During wrist movements under no-load conditions, the corresponding estimation of muscle forces was denoted as initial muscle force *F*_0_. Under the external loads, the wrist additionally bore the gravitational force *G* of the weight and the inertial force *F*_inertia_ generated by the wrist itself. Hence, the dynamic muscle force *F*_dynamic_ of the wrist could be expressed as follows:

(9) $$F_{\mathrm{dynamic}}=F_{0}+G+F_{\mathrm{inertia}}$$

where *G* = *mg*, *m* is the mass of the weight, *g* is the gravitational acceleration, $F_{\mathrm{inertia}}=mr\ddot{\beta }$, *β* denotes the F/E angle, *r* is the distance from the rotation center of the wrist to the force *G*, and *r* = 70 mm.

To evaluate the performance of the movement recognition algorithm and the muscle force estimation model under the resistance rehabilitation, a performance experimental platform was constructed in accordance with the established rehabilitation strategy.

## 3. Results

### 3.1. Verification of the Movement Recognition Algorithm

**Figure 5:**
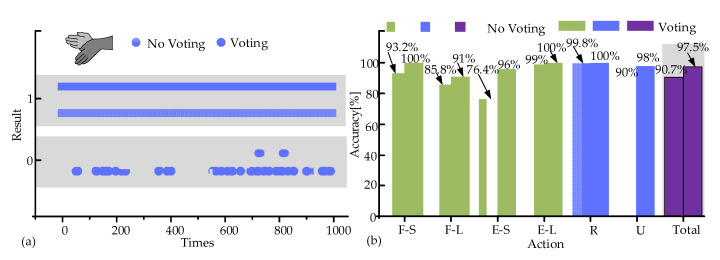
Recognition accuracy with voting decision. (**a**) Results of ulnar deviation. (**b**) Accuracy of six movements.

To verify whether the voting decision enhanced the accuracy of the movement recognition algorithm, the experiments were conducted under six typical movements. Three volunteers were informed of the specific movements to be performed, with each movement repeated 1000 times. The wearing position on the volunteers was cleaned with alcohol. The recognition results of ulnar deviation with/without the voting decision were recorded, as shown in Figure 5a, where 0 and 1 represent incorrect and correct results, respectively. The corresponding accuracy of the two types of results was calculated (Figure 5b). The accuracy was improved for all six movements with the average accuracy increasing from 90.7% to 97.5%. The U and E-S movements exhibited the greatest improvement in accuracy. The results indicated that there was a significant enhancement in the accuracy of movement recognition after adding the voting decision into the recognition algorithm.

**Figure 6:**
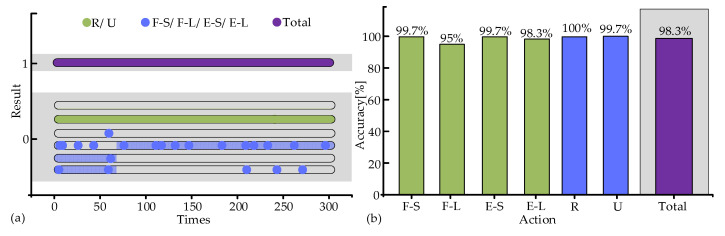
Recognition accuracy of movement recognition algorithm. (**a**) Results of six movements. (**b**) Accuracy of six movements.

To further validate the recognition accuracy after adding the intensified algorithm, volunteers repeated the movements as in the previous experiments. Five sets were completed for each movement, with 60 repetitions per set, for a total of 300 repetitions. A total of 1800 recognition outcomes were produced, where 23 outcomes were incorrect (R 0, U 1, F-S 1, F-L 15, E-S 1, E-L 5), as shown in Figure 6a. The accuracy of each movement had been further improved, and the overall accuracy of the six movements was 98.7% (Figure 6b).

### 3.2. Muscle Force Estimation

**Figure 7:**
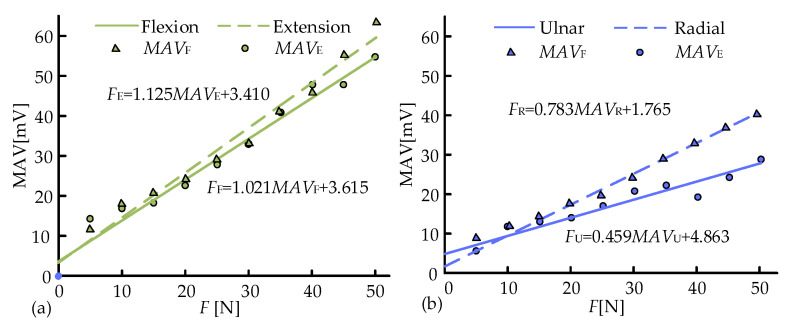
Muscle force estimation model of (**a**) F/E and (**b**) R/U.

The *MAV* of the sEMG signals under a certain external load and movement was obtained through calculating the absolute value of each data point, adding up these absolute values, and dividing the sum by the number of data points. *MAV* was taken as the eigenvalues of this estimation model. Since all the measurement experiments were performed three times, there were three *MAV*s, and their average value was plotted in Figure 7. It could be observed that the data points had a good linear relationship. The data of average *MAV*s were then linear fitted by MATLAB R2020a to derive the estimation model for F/E and R/U:

(10) $$\left\{\begin{array}{l} F_{E}=1.125MAV_{E}+3.410\\ F_{F}=1.021MAV_{E}+3.615\\ F_{R}=0.783MAV_{R}+1.765\\ F_{U}=0.459MAV_{U}+4.863 \end{array}\right.$$

where the subscripts of *MAV* were the initials of corresponding movements.

**Figure 8:**
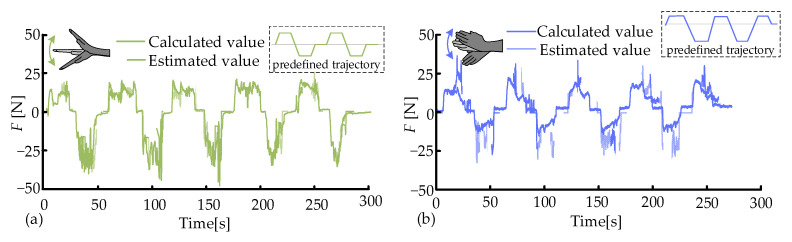
Estimated and actual muscle forces of (**a**) F/E and (**b**) R/U.

The established model (10) was deployed in the control system of PWRR to estimate the muscle force of the wrist during the resistance training. The volunteers tried to move along the predefined trajectory. With the aid of the estimation model and the force sensor Nano25, the estimated and actual muscle forces were obtained in real time, as shown in Figure 8. The two force curves exhibited similar trends. However, the estimated forces were lower than the actual ones in the F/E movement, and the relationship was opposite in the R/U movement. The RMSE of the two forces was calculated, where the average RMSE values of F/E and R/U were 4.2 N and 5.8 N, respectively. Some RMSE values deviated significantly from the average value, possibly due to the following: (1) most of the deviation values were caused by the minor discontinuous movements during the resistance training and (2) errors were introduced by the mounting method of the force sensor and the frictional resistance.

**Figure 9:**
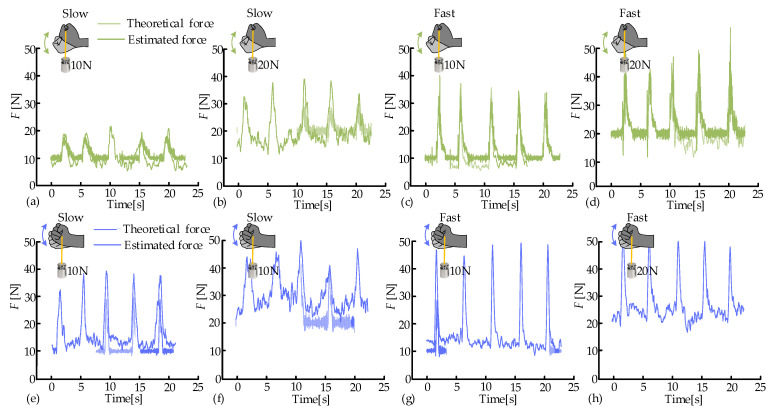
Theoretical and estimated forces of flexion (**a**–**d**) and radial deviation (**e**–**h**).

**Figure 10:**
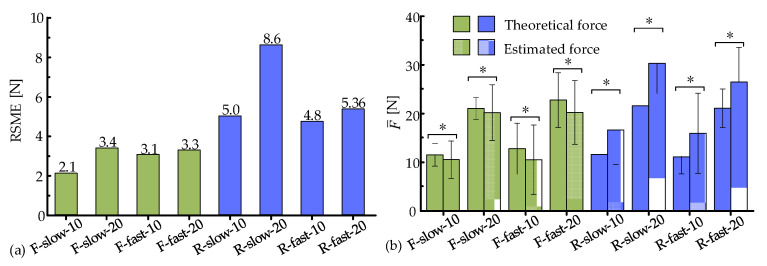
Evaluation of muscle force estimation. (**a**) RMSE and (**b**) average values $\bar{F}$ of the theoretical and estimated forces (The asterisk * was significance, *p* < 0.05).

To further assess the accuracy of the estimation model (9), the flexion movements were performed under the slow/fast modes and two external loads (10 N, 20 N). The calculated results from Equation (9) were taken as the actual values, where the angular acceleration $\ddot{\beta }$ of F/E was measured by the VICON system. The estimated values were obtained by the estimation model (10) and the *MAV*s of collected sEMG signals. The calculated and estimated values are presented in Figure 9a–d. The estimated and calculated muscle forces exhibited similar trends and had almost no phase difference. However, during the static phase, the estimated values were relatively stable and slightly lower than the calculated values. Their relationship was opposite at the peak positions. The muscle force estimation model possessed an overall high accuracy. As the external loads increased, the peak values of the estimations significantly rose due to the inertia of the weights. Similarly, the peak values also increased notably with the increase in flexion speeds. The estimation accuracy was optimal at the condition of F-low-10, with an average RMSE of 2.1 N (Figure 10a). The RMSE values under the remaining three experimental conditions were similar (3.4 N, 3.1 N, and 3.3 N, respectively). Nonetheless, the relative estimation accuracy was higher in the fast mode.

Similarly, the radial deviation movements were performed under the slow/fast modes and two external loads (10 N, 20 N). The calculated and estimated values exhibited similar trends and had almost no phase difference, as depicted in Figure 9e–h. However, during the static phase, the estimated forces were slightly higher than the calculated ones, and the peaks of the estimated forces were slightly higher. A possible reason was that radial deviation movements primarily rely on the synergistic activation of muscles, and the amplitude of this movement was relatively small. It was interesting to notice that the speeds and external loads had similar impacts on muscle activation in both flexion and radial deviation movements. The estimation accuracy was optimal at the condition of R-fast-10, with an average RMSE of 4.8 N (Figure 10a). The RMSE values under the remaining three experimental conditions were similar (5.0 N, 8.6 N, and 5.36 N, respectively). However, the estimation accuracy of the radial deviation movement was lower than that of the flexion movement.

The average values $\bar{F}$ of estimated and calculated forces under different experimental conditions were calculated to thoroughly assess the estimation accuracy, as illustrated in Figure 10b. During flexion movements, the estimated values were generally lower than the actual values, and their relationship was opposite during radial deviation movements. As the speeds and external loads increased, the absolute difference between the two forces tended to enlarge, while the relative accuracy was improved. It is worth noting that the speeds had little impact on the average values $\bar{F}$ in the movements of flexion and radial deviation.

Furthermore, the accuracy of flexion movements was significantly better than that of radial deviation movements, with the highest relative accuracy of 4.26% under the F-slow-20 condition. That was primarily attributed to the larger range of motion and the concentrated activation of the involved muscles. The result indicated that the constructed estimation model, with its simplicity and real-time capability, was capable of muscle force estimation in rehabilitation training.

## 4. Discussion

This study presented an sEMG signal-based control algorithm for PWRR. By utilizing the movement recognition algorithm and muscle force estimation model, real-time analysis of EMG signals and precise control of PWRR were achieved. To construct the movement recognition algorithm, raw sEMG signals were collected for seven distinct wrist movements. Time-domain eigenvalues were then extracted from these signals. The eigenvalues were selected after the extraction to establish a dataset for classifying wrist movements. Subsequently, a BPNN model was developed using the PyTorch 2.0 framework. After validation with the test set, the model demonstrated a classification accuracy of 97.4%. However, the model’s accuracy decreased in real-time practices due to the inconsistency of EMG sensor placements and the non-standardization of wrist movements. A voting decision mechanism was proposed to enhance accuracy. Additionally, an intensified algorithm was also proposed, which could serve as a triggering condition for the control of PWRR. The static and dynamic models of the muscle force estimation were established to describe the relationship between *MAV*s and muscle forces. A resistive rehabilitation could be achieved by using these models.

Currently, most wrist rehabilitation robots primarily rely on preset programs to guide patients through simple, repetitive rehabilitation exercises, failing to adequately consider the patients’ engagement or integrate muscle activities into the control system. Compared with the wrist rehabilitation robots developed by Cappello [11] and Spencer [12], sEMG signals were utilized as control inputs, aiming to effectively enhance patients’ proprioception, thereby significantly improving the fun and engagement of patients in the rehabilitation process. For wrist movement recognition, most studies have focused on constructing classification models, with fewer proposing optimal methods for real-time recognition and schemes for applying the models to the actual robot control. Tsujiuchi et al. [23] developed a method capable of recognizing seven types of hand movements with a recognition accuracy rate of 90%. Kaichi et al. [19] constructed a model capable of recognizing 53 types of gestures with an accuracy rate of 60%. Khokhar et al. [26] developed a wrist robot controlled by EMG signals, with an average accuracy rate of 97.4% for four wrist movements. Rechy-Ramirez et al. [27] developed a wrist recognition system that detected five wrist motion actions (relaxation, extension, pronation, supination, flexion) through leap motion controllers and MYO armbands, with an accuracy rate of 85% for the five movements. The BPNN model constructed in this study had an accuracy rate of 97.4%. With the help of a voting decision mechanism and an intensified algorithm, the accuracy of movement recognition in the wearing status of different volunteers was further enhanced to 98.7%. The accuracy of motion recognition and muscle force estimation was able to significantly reduce the latency of instructions. PWRR did not experience stuttering or delays, and it could promptly execute the predefined rehabilitation exercises, avoiding operation errors caused by delays. This enabled patients to feel as if they were in a real environment, enhancing the sense of immersion and the naturalness of interaction and thus improving the rehabilitation outcomes.

There existed a strong linear correlation between the muscle strength and muscle activation. Such a linear relationship had previously been verified in various situations [25,26]. Yu et al. [25] completed a linear regression between the first two principal components of extracted features and the recorded wrist torques, validating the effectiveness of the proposed approach. In the literature [42], the wrist torque was estimated by decomposing the sEMG signals. When processing the electromyography signal data under the activation condition of motor units, a linear interpolation was used to deal with the noise and discontinuities in the signals, so as to improve the quality and stability of the data. Since missing points would occur acquisition process of EMG signals, linear interpolation was used to deal with the missing values in the EMG signals, so as to ensure the integrity of the data [23]. Chen et al. [43] proposed a musculoskeletal model and algorithm based on sEMG signals to estimate the wrist torque. When dealing with the data of motor unit discharges, the linear interpolation was used to process the data in order to fill in the missing values or smooth the data. Similarly, the established muscle force model was only linearly related to the *MAV*s, and this model had a high degree of credibility. In order to verify its evaluation accuracy, sEMG signals under different external loads and velocities were measured in a dynamic environment. The calculated and estimated values exhibited similar trends and had almost no phase difference.

Due to the current limitations of wrist rehabilitation devices and sEMG applications, we introduced a novel control algorithm based on movement recognition and muscle force estimation. This control algorithm provided innovative perspectives and solutions for the intelligent control of rehabilitation robots. Nonetheless, there existed certain limitations: (1) The generalizability of the movement recognition algorithm and muscle force estimation model needed to be further verified, since the sEMG signals as the inputs were collected from healthy individuals. (2) Although actual muscle forces could be estimated from sEMG signals to achieve resistive rehabilitation, there existed relatively small errors in the estimated results. The estimation models were primarily constructed using experimental data under a static environment, considering only the impact of loads on forearm sEMGs without fully accounting for the changes caused by muscle contractions during the wrist flexion or radial deviation. (3) The wearing position of the sensor will affect the prediction accuracy, even though the sensor is equipped with an inertial unit inside. Therefore, the sensor should be worn in the same position as much as possible, and the sensor should maintain an approximate posture according to the signal of the inertial unit. To address the aforementioned shortcomings, transfer learning algorithms could be studied in depth to build a universal movement recognition model in the future. Additionally, wrist angle variables could be introduced to refine the muscle force estimation model, thereby enhancing the accuracy and practicality.

## 5. Conclusions

The movement recognition algorithms and the muscle force estimation model were proposed based on the forearm sEMGs, with the movement recognition accuracy rate reaching 98.7%. Additionally, under the resistive mode, the estimated force curves were largely consistent with the actual ones. There existed relatively small errors in the estimated results under different loads and velocities. The established algorithms could be applied to the control system of the wrist rehabilitation robot. This approach enhances patient engagement and proactivity, providing a significant reference for the optimization of future control strategies.

## Data Availability

Data will be made available on request.

## Abbreviations

The following abbreviations are used in this manuscript:

sEMG	Surface electromyography
CNN	Convolutional neural network
HD-EMG	High-density electromyography
SAE-DNN	Stacked autoencoders and deep neural network
BPNN	Backpropagation neural network
*MAV*	Mean absolute values
*WL*	Waveform length
*ZC*	Zero crossing
*VAR*	Variance
*RMS*	Root mean square
NMF	Non-negative matrix factorization
